# Vacinação antivariólica na província do Paraná, 1853-1863

**DOI:** 10.1590/S0104-59702024000100064

**Published:** 2024-12-16

**Authors:** João Pedro Dolinski

**Affiliations:** i Doutor, História Social/Universidade Federal do Paraná. Curitiba – PR – Brasil joaodolinski@gmail.com

**Keywords:** Circulação, Varíola, Vacina antivariólica, Província do Paraná, Circulation, Smallpox, Smallpox vaccine, Paraná, Brazil

## Abstract

This study reflects on the efforts to disseminate and administer the smallpox vaccine in the Brazilian province of Paraná between 1853 and 1863, taking a circulation-based perspective. Chronologically, the first milestone is the founding of Paraná and the second is the drafting of the first regulations for vaccination in the province. This research draws on the assumptions of translocal microhistory and the theoretical conceptions expounded by Kapil Raj and Fa-ti Fan. The central argument is that the factors blocking the spread of the smallpox vaccine were the very processes by which vaccination was circulated, and that popular resistance to the prophylactic agent, itself part of these processes, was not widespread.

O objetivo deste artigo é examinar os esforços de aplicação e generalização da vacina antivariólica no Paraná entre os anos 1853-1863 a partir de uma perspectiva circulatória. De maneira mais específica, investigamos, em um primeiro momento, as opiniões de médicos, presidentes de província e ministros do Império a respeito do fracasso da vacinação no país e suas sugestões para reverter esse quadro. Em seguida, analisamos o surto de varíola ocorrido na cidade de Paranaguá em 1863. Por fim, buscamos realizar uma reflexão inicial sobre as estatísticas dos vacinados na província do Paraná, elaboradas pelo médico José Cândido da Silva Muricy (1827-1879). A primeira baliza cronológica representa o ano de fundação da província paranaense. Por sua vez, a segunda diz respeito ao ano em que foi elaborado o primeiro regulamento para a vacinação no Paraná.

O argumento central deste estudo afirma que as dificuldades de difusão da vacina no Paraná, assim como no Brasil imperial, eram especificidades intrínsecas aos processos de circulação que caracterizavam de forma singular os serviços de vacinação no Brasil do século XIX. Entre essas dificuldades, destacamos a infraestrutura viária e os recursos técnicos, humanos e econômicos que, conforme a região do país, eram mínimos ou simplesmente não existiam. Outro obstáculo, bastante enfatizado pela documentação primária e pela historiografia, era a resistência popular. Afirmamos que ela não era um fenômeno generalizado. Durante as epidemias no Paraná, muitas pessoas pressionavam os vacinadores para receber o imunizante. Além disso, como veremos adiante, a própria resistência popular constituía um dos aspectos dos processos de circulação.

Ao definir circulação, Kapil Raj sublinha o seu caráter de transformação. Ela se caracteriza, segundo o autor, pelas mudanças que os conhecimentos e as práticas passam ao deslocarem-se pelo interior de espaços específicos, com regras codificadas que favorecem, ampliam ou dificultam as conexões entre lugares, grupos ou indivíduos conforme as conjunturas históricas, políticas, econômicas e sociais (Duarte, jun. 2016). Por outro lado, Kapil Raj (2013) reconhece que os processos de circulação também denotam as relações de poder, as resistências e as negociações inerentes às interações interculturais. Em virtude disso, a resistência popular à vacina é compreendida aqui como um dos fatores que integram os processos de circulação. Tais aspectos, em conjunto com as observações de Fa-Ti Fan (2012), revelam como esses processos não são fluidos, estáveis e uniformes. Eles apresentam *nuances* em função das particularidades locais. Não interpretamos essas *nuances* como algo negativo. Certamente, existiam limitações e obstáculos à generalização da vacina apontados pela documentação primária. Entretanto, ao seguirmos a abordagem de Raj e Fan, essas barreiras não serão pensadas como problemáticas, mas como aspectos singulares que conformavam uma caracterização local dos próprios processos de circulação.

Do ponto de vista da micro-história translocal (De Vito, 2020), lugares ou regiões não seriam mônadas isoladas ou zonas neutras, mas sim pontos de cruzamento de múltiplas conexões em todas as escalas, com suas próprias dinâmicas relacionais internas e externas. Nessas circunstâncias, artefatos, indivíduos, práticas e saberes circulam de formas distintas, segundo as particularidades e as conjunturas que caracterizam os diferentes espaços. Diante disso, compreendemos o conceito de circulação não apenas no sentido de troca, apropriação, ressignificação ou conexão, mas, principalmente, como distribuição. Ao focar nesse aspecto, buscamos esclarecer como os recursos técnicos, científicos, financeiros e humanos, necessários tanto à prática médica e sanitária como à própria generalização da vacina antivariólica, circulavam no interior do Brasil imperial de maneira complexa e não linear.

Dilene Raimundo do Nascimento (2005) afirma que a doença, como objeto de estudo, possibilita o conhecimento sobre estruturas e mudanças sociais. A varíola, portanto, não seria apenas um fenômeno patológico, mas também social, político, cultural e econômico. Como veremos ao longo deste estudo, ela revela perspectivas peculiares da sociedade imperial, mais especificamente, de sua infraestrutura e organização administrativa.

A historiografia da varíola é extensa e apresenta um quadro de reflexões bastante diverso do ponto de vista das abordagens e dos referenciais teórico-metodológicos.^1^ Em função disso, os critérios que estabelecemos para selecionar os trabalhos que destacamos a seguir foram a convergência com o recorte espacial e as relevantes contribuições que podem fornecer para o aprofundamento das investigações propostas por este artigo. É importante ressaltar que esses trabalhos produziram análises a partir de perspectivas teóricas e metodológicas distintas daquelas adotadas nesta pesquisa, o que não inviabiliza o nosso objetivo de ampliar o diálogo e complementá-lo, contribuindo para o avanço do debate historiográfico dedicado à varíola.

Embora não fique adstrito, o trabalho de Fábio Kühn e Jaqueline Hasan Brizola (2019) ancora-se na questão cultural para compreender a resistência à vacina. Os autores chegam a mencionar a circulação de pessoas (soldados) como fator importante para a disseminação da varíola no Rio Grande do Sul. Apresentam as estatísticas sobre os infectados, mas, em função das suas opções teóricas e metodológicas, não problematizam a relação desse saber com o desenvolvimento e a modernização dos instrumentos de administração do Estado.

Com uma linha de análise congênere, Oliveira (2003) compreende a resistência popular à vacina a partir do avanço da modernidade e dos seus impactos sobre as antigas tradições e costumes de segmentos sociais espezinhados. Em relação às questões de circulação, Oliveira argumenta que, até o século XX, a varíola não tinha afetado Goiás em função do isolamento daquele espaço. Entretanto, com o desenvolvimento do progresso, mais especificamente das ferrovias e dos processos migratórios, os surtos de varíola teriam aumentado na região Centro-Oeste.

Com um eixo analítico distinto, Hochman e Souza (2022) examinam as dificuldades do poder público da Bahia em aplicar e difundir a vacina antivariólica. Os autores trabalham com os relatórios de presidentes da província da Bahia e com dados estatísticos de vacinados publicados em periódicos locais. A partir disso, discutem, entre outras questões, as percepções e ações das autoridades públicas em relação ao processo de produção, conservação, distribuição e aplicação das vacinas. Muitos dos problemas constatados pelos autores na Bahia também foram observados por nós no Paraná.

Liane Maria Bertucci (2019) apresenta subsídios empíricos importantes que ajudam a corroborar nosso argumento sobre a resistência popular à vacina. A autora trabalha com um conjunto documental que também foi utilizado nesta pesquisa, destacando, inclusive, as mesmas questões aqui observadas, como, por exemplo, as dificuldades de distribuição da vacina e a relação entre o conhecimento médico e a estatística. No entanto, as interpretações de Bertucci divergem das nossas. Nesse aspecto, este trabalho pretende colocar em relevo questões não discutidas ou não aprofundadas pela autora.

O conjunto de fontes utilizado para esta pesquisa é composto por relatórios provinciais, ministeriais e médicos; decretos federais; estatísticas médicas e regulamentos sanitários. Trata-se de uma documentação de caráter oficial, científico e serial que denota o ponto de vista de uma elite política e intelectual brasileira do século XIX. Os documentos utilizados nesta pesquisa encontram-se disponíveis no Arquivo Nacional, na Biblioteca Pública do Estado do Paraná e no *site* do Center for Research Libraries.

## Os serviços de vacinação na província do Paraná

José Antonio Vaz de Carvalhaes, vice-presidente da província do Paraná, considerava ineficaz o serviço de vacinação na região. Em sua opinião, a razão não estava na falta de pus vacínico (importado da Real Sociedade Jenneriana de Londres), mas sim no baixo número de vacinados, que, em 1857, totalizou 462 pessoas em toda a província (Carvalhaes, 1858).^2^ Na Corte, esses e outros problemas vinham desde o tempo da Junta da Instituição Vacínica. Em 1834, o então ministro do Império, Antônio Pinto Chichorro da Gama, expôs a desorganização da junta, os baixos salários pagos aos seus funcionários e propôs a sua transformação em órgão central, além da criação de juntas provinciais de vacinação (Gama, 1834).

Súplicas pelo estabelecimento de juntas provinciais também foram feitas por Bernardo Pereira de Vasconcellos (1838), ministro dos negócios do Império. De acordo com ele, uma comunicação mais eficiente entre a junta central e as juntas provinciais, e a formação de um quadro estatístico mais rigoroso possibilitariam uma regularização mais efetiva, além de proporcionar maior segurança na aplicação do imunizante. Porém, tentativas de sistematização dessa atividade em nível nacional ocorreriam somente em 1846, com a regulamentação do Instituto Vacínico da Corte e a criação dos cargos de comissários vacinadores provinciais, municipais e paroquiais.

Luiz Pedreira do Coutto Ferraz, ministro dos negócios do Império, pôs em evidência outros entraves relativos aos serviços de vacinação. Lotes de pus vacínico vinham se deteriorando em razão do penoso procedimento de importação e distribuição. Faltavam ainda pessoas comprometidas com a propagação da vacina. Isso podia ser atribuído, de acordo com o ministro, à desmotivação por parte dos vacinadores municipais e paroquiais em função da ausência de gratificações e pagamentos pelos serviços (Ferraz, 1855). Além disso, o ministro nos deixa escutar o seguinte:

Em um país de vastíssima extensão, como é o nosso, onde a população se acha disseminada em grupos, em muitos pontos separados uns dos outros por grandes distâncias e péssimas estradas; onde, portanto, é assaz difícil levar a instrução necessária para convencer os habitantes de seus verdadeiros interesses em muitos respeitos; onde também dificilmente pode chegar a ação da autoridade; já para os prontos socorros que é mister prestar a todos os habitantes, já para aplicar a ação coercitiva contra aqueles que não se quiserem sujeitar a certas medidas, que é do rigoroso dever do governo empregar a bem da saúde pública; só o tempo, o aumento da população, e os progressos da civilização podem destruir preconceitos antigos e inveterados e generalizar os benefícios de certas instituições (Ferraz, 1855, p.11).

Como podemos ver, as queixas sobre a resistência generalizada da população perseveravam, demonstrando as discrepâncias entre universos mentais distintos a respeito das concepções de saúde e doença. As observações do ministro ainda ressaltam as dificuldades de expansão dos serviços médicos no país em virtude da extraordinária extensão geográfica. Para endossar as lamentações de Coutto Ferraz e demonstrar como essas e outras questões também estavam presentes no Paraná, ressaltamos as palavras de José Antonio Vaz de Carvalhaes (1857, p.43-44):

Custa a crer, Srs., mas é verdade que são bem poucos os que na província recebem esse preservativo, tão fácil quão maravilhoso, contra uma enfermidade terrível, que, quando não mata, quase sempre mutila e desfigura. Na impossibilidade de imputar o mau estado deste ramo de serviço ao desmazelo do vacinador provincial, de cuja inesgotável atividade e incansável zelo sou eu o primeiro a dar consciencioso testemunho, devo crer na informação que me dá, de que nem resposta recebe aos multiplicados pedidos de fluido vacínico, que tem dirigido ao respectivo instituto.

Por sua vez, o presidente da província do Paraná, José Francisco Cardoso, afirmava que: “Sem disposições eficazes e corretivas, não creio que o serviço da vacinação prospere, e menos que ofereça resultados benéficos. As nossas municipalidades, a quem cabe a comunicação de penas e a iniciativa de posturas, de mãos dadas com a autoridade policial, poderiam coagir os encarregados da infância a reprimir tais faltas” (Cardoso, 1860, p.61). Cardoso não foi a única autoridade a expressar a necessidade de medidas mais enérgicas (leia-se autoritárias) para a generalização da vacina antivariólica. Dois anos após a publicação do seu relatório, o presidente Antonio Barbosa Gomes Nogueira (1862, p.42) fez a seguinte observação:

A falta, às vezes, de fluido vacínico, o modo pouco filantrópico e patriótico com que se conduzem alguns dos vacinadores paroquiais e mais ainda a relutância que a população oferece à vacinação, em cujos salutares efeitos descrê, são outros tantos motivos que justificam o minguado número de vacinados. Isto posto, é fácil de conceber que a coação é um dos meios por que poder-se-á obter melhores resultados desse serviço, nas circunstâncias atuais. Para a consecução, portanto, de semelhante fim, torna-se indispensável que as penas marcadas nos códigos de posturas municipais sejam impostas com severidade aos pais e tutores que furtarem seus filhos pupilos ao benefício que oferece o preservativo da varíola.

Era praticamente consenso entre as autoridades públicas brasileiras daquela época (1840-1860) que um dos problemas relativos à generalização da vacina era a resistência popular. Acreditavam que uma intervenção mais coercitiva no corpo social poderia solucionar essa questão. A despeito disso, o ministro Antônio Pinto Chichorro da Gama sublinhou outros filamentos dessa intrincada trama, como os baixos salários, a desorganização dos serviços de vacinação e a falta de uma estrutura sanitária mais coesa. Para o ministro Bernardo Pereira de Vasconcellos, a questão central seria, além da ignorância popular, a falta de quadros estatísticos mais rigorosos sobre a vacinação da população. Por seu turno, Luiz Pedreira do Coutto Ferraz assinalou a deterioração dos lotes de pus vacínico em virtude do processo de importação e distribuição.

Como podemos observar, o combate à varíola no Brasil oitocentista mediante a aplicação da vacina apresentava contornos específicos em função de fatores inerentes aos processos de circulação. O caráter singular desses processos pode ter contribuído ainda para reforçar a desconfiança popular em relação à vacina. Como a linfa importada de Londres não resistia até a chegada aos locais de vacinação, o imunizante aplicado não surtia o efeito esperado, e isso dava azo à descrença da população nas prescrições da medicina acadêmica.^3^ Além disso, relações de poder (como forçar a população a se vacinar) e resistências (a desconfiança popular em relação à vacina), conforme observado por Raj (2013), são inerentes aos processos de circulação. É importante ressaltar que a resistência popular não ocorria por ignorância dos segmentos subalternos da sociedade. As camadas sociais inferiores buscavam resolver seus problemas de saúde junto aos curandeiros, barbeiros e benzedores em função dos seguintes fatores: esses terapeutas populares possuíam um conhecimento ancestral a respeito das doenças; eles compartilhavam com essa população um mesmo universo mental e social; e tinham um vocabulário acessível e compreensível para os enfermos, distinto daquele vocabulário técnico-científico utilizado pelos médicos acadêmicos.^4^ Não obstante, a resistência ao imunizante não era um fenômeno generalizado.

No que diz respeito à importação e distribuição da vacina, trata-se, sem dúvida, de questões relacionadas à infraestrutura viária e à obtenção de recursos. Nessas circunstâncias, se a categoria de circulação for pensada como ferramenta descritiva no sentido mais direto, esses aspectos poderão ser interpretados como impedimentos que limitavam a circulação. Entretanto, a categoria de circulação é utilizada aqui como ferramenta metodológica, e, portanto, esses obstáculos são vistos como parte dos processos de circulação, uma vez que estes não são homogêneos nem lineares. Esses percalços não são simplesmente problemas de caráter negativo. Eles sinalizam para uma singularidade do Brasil imperial quanto aos serviços de vacinação antivariólica no século XIX.

Na próxima seção, discutiremos como esse aspecto singular dos processos de circulação no Brasil imperial caracterizou os serviços de vacinação na cidade de Paranaguá. Essa singularidade pode ser constatada empiricamente na atuação de médicos e do poder público durante os surtos de varíola que atingiram a região. Apesar de ser uma cidade secundária, Paranaguá abrigava o principal porto da província paranaense, e, segundo Hobsbawm (2014, p.31), “estar perto de um porto era estar perto do mundo”.

## O surto de varíola de 1863 em Paranaguá

Em um relatório enviado ao governo da província do Paraná, o médico Alexandre Bousquet observou que o surto de varíola ocorrido na cidade de Paranaguá em 1863 vitimou, em sua maioria, indivíduos pertencentes às classes sociais inferiores. Conforme o relatório, essas pessoas apresentavam maior suscetibilidade às bexigas em função das “infecções físicas constitucionais” e de moléstias como a bouba. A principal forma de propagação da varíola, segundo Bousquet, foi a lavagem de roupas em fontes onde inexistia critério de separação entre peças utilizadas por pessoas doentes e sadias. Ele conjecturou que aproximadamente quatrocentas pessoas teriam sido afetadas pela varíola entre 1862 e 1863 naquela cidade. Somente em sua clínica, foram diagnosticados 133 casos nesse período (Bousquet, 27 jul. 1863). Entre março e maio de 1863, 85 pessoas adoeceram, das quais 13 desceram à sepultura (Pinto, 1863). Por sua vez, o relatório do governo da província do Paraná apontou 695 pessoas acometidas pela varíola em Paranaguá, das quais 85 faleceram (Silva, 1864).

Em 1854, a província do Paraná contava com aproximadamente 62 mil habitantes. Nesse mesmo período, Paranaguá tinha uma população estimada em 6.533 pessoas (Vasconcelos, 1854). Se observarmos o mapa da vacinação praticada no Paraná, veremos que entre 1857 e 1863 foram vacinadas, em toda a província, 3.435 pessoas, das quais 250 apenas na cidade de Paranaguá. Esses dados indicam o ínfimo número de vacinados na província. Conjecturamos que o número de infectados também fosse baixo. Questões relacionadas a resistência popular, infraestrutura viária e recursos técnicos, humanos e financeiros ajudam a compreender a reduzida taxa de imunizados. Por outro lado, a própria infraestrutura viária, isto é, a sua exiguidade, bem como a dispersão populacional e a extensão geográfica da região, possibilita compreender o baixo número de infectados. Em substância, os processos de circulação configuravam o quadro geral da varíola e da vacinação na província do Paraná.

**Tabela 1:** : Mapa da vacinação praticada na província do Paraná, 1857, 1862 e julho a dezembro de 1863*

Sexo		Condição		Resultado da vacinação			Total
masc.	fem.	livre	escravos	Vacinação regular	Sem resultado	Não foram observados	
1.934	1.457	2.974	417	3.003	229	203	3.435

* População da província do Paraná em 1858: 69.380 pessoas.

Fonte: Carvalhaes (1858); Mattos (1859); Nogueira (1863); Silva (1864).

Em relação à origem do surto em Paranaguá, algumas controvérsias puderam ser observadas entre o Ministério do Império e os médicos Alexandre Bousquet e Olegário Cesar Cabussú. Bousquet chegou à cidade de Paranaguá 15 dias após a manifestação dos primeiros casos e logo passou a coletar informações junto à população. No dia 1º de março, atracara no porto um iate procedente de Laguna (SC), carregado com farinha de mandioca e milho. A bordo do iate, fechados no porão, estavam dois indivíduos acometidos pela varíola. As cinco pessoas incumbidas de descarregar o iate foram contaminadas, e uma delas veio a falecer pouco tempo depois. Pressionado pela população para administrar o imunizante (o que destoa da típica resistência à vacina), o vacinador municipal precisou recorrer à variolização em virtude do estado de deterioração da linfa importada. Após a inoculação de algumas crianças, o médico argumentou que os sinais apresentados por elas foram decorrentes da variolização,^5^ distintos daqueles típicos sintomas atenuados da vacinação. Confuso e forçado a acalmar os ânimos populares, Bousquet mudou seu diagnóstico e afirmou tratar-se de febre eruptiva variólica, isto é, bexigas naturais. Independentemente disso, os casos foram aumentando e espalhando-se com grande rapidez. De acordo com Bousquet, antes de sua chegada à cidade, existiam dez ocorrências da moléstia. Após a variolização, surgiram 25 novos casos (Bousquet, 6 abr. 1863). Nas palavras de Alexandre Bousquet (citado em Cabussú, 23 abr. 1863):

O pus vacínico do comissariado desta cidade era inativo, como quase sempre acontece: o nosso vacinador, instado pela população para subministrar-lhe um preservativo contra a propagação possível das bexigas, de que já tinha havido alguns casos, teve a infeliz lembrança de substituir o pus variólico de um de seus doentes, ao pus vacínico que faltava e o inoculou em várias crianças nos domingos 8 e 15 de março. No dia 16 encontrei 15 crianças com febre, delírios, vômitos, cefalalgia, sintomas que atribuí logo aos efeitos da vacinação.

Ao chegar em Paranaguá, Bousquet deparou-se com um quadro dramático. Não havia linfa em bom estado de conservação, o que forçou o vacinador municipal a adotar o método já superado da variolização. As peculiaridades inerentes aos processos de circulação no Brasil imperial impunham desafios excruciantes ao enfrentamento das doenças epidêmicas que grassavam àquele tempo. Paranaguá era uma cidade portuária; todavia, as linfas importadas de Londres chegavam somente ao Rio de Janeiro. Para dificultar ainda mais, as instruções da *cowpox* vinham em idioma inglês. Além disso, não havia uma infraestrutura minimamente constituída, com ferrovias e rodovias, capaz de assegurar a distribuição da vacina.

A construção de estradas de ferro era um empreendimento que demandava o investimento de grandes somas de capital. Mesmo na Inglaterra, a construção de ferrovias não foi um processo simples e fácil. Além da enorme quantidade de dinheiro, havia a necessidade de extrair toneladas de ferro e carvão. Cada milha de linha exigia trezentas toneladas de ferro apenas para os trilhos (Hobsbawm, 2014). A malha ferroviária no Brasil imperial não era suficiente para cobrir a enorme extensão geográfica do país.^6^ No Paraná, as poucas estradas que existiam não permitiam uma circulação rápida e segura em função de suas condições precárias.

Além da falta de uma rede de ferrovias e rodovias, as dificuldades em produzir a vacina no país também obstaram os esforços das autoridades públicas e sanitárias na aplicação e difusão do imunizante. Tentativas de obtenção da *cowpox* verdadeira no Brasil datam da década de 1830, com as tentativas do doutor Capistrano de renovar a vacina mediante a inoculação de novilhas. Contudo, em função da inexistência de recursos técnicos locais, a regeneração da linfa só foi possível a partir da década de 1880. Para tentar minimizar o problema, o governo imperial importava a linfa já humanizada da Europa, acondicionada em lâminas de vidro, tubos capilares ou recipientes de chumbo. Mesmo assim, grande parte do material já chegava ao Rio de Janeiro deteriorado, e, caso fosse distribuído por via marítima, pouquíssimos lotes chegariam em bom estado de conservação às demais províncias. A reprodução da linfa importada também era difícil, uma vez que os lancetados recusavam-se a retornar para a extração do pus. Havia também outra questão, qual seja, a vacina não garantia imunização permanente. A cada dez anos, os indivíduos necessitavam ser revacinados, o que contribuía para o descrédito em relação à eficácia do imunizante (Chalhoub, 1996).

Olegário Cesar Cabussú, primeiro cirurgião do Corpo de Saúde, criticou os serviços sanitários prestados durante o surto de varíola em Paranaguá. Em sua visão, o médico e o secretário encarregados da visita ao porto não realizaram um exame minucioso do iate, nem sequer verificaram se trazia carta limpa, documento que atestava a ausência de doenças epidêmicas a bordo. Os indivíduos responsáveis pela descarga do iate buscaram tratamento em terra, onde doentes e sãos conviviam em total promiscuidade nos diferentes espaços de cura. A embarcação não foi posta em quarentena, permitindo-se a livre circulação e comunicação entre os doentes do barco e a população. A doença inicialmente manifestou-se de maneira discreta, levantando suspeitas em relação ao diagnóstico de bexigas. Para Cabussú, a existência de erupções na pele bastava para a confirmação da moléstia. Em sua perspectiva, as principais causas do surto foram a falta de visita médica rigorosa e a incapacidade de Bousquet e do vacinador municipal em diagnosticar a bexiga logo nos primeiros casos. Confirmada a doença em seus primórdios, ações básicas para proteger a população de um surto epidêmico, como a separação de doentes e a vacinação, seriam facilmente postas em prática, segundo Cabussú.

Algumas pessoas relataram que os vacinados retornavam a seus lares com as vestes ensanguentadas, indicando que o procedimento fora realizado equivocadamente. Para o cirurgião do corpo de saúde, o vacinador municipal agia com má vontade. Em sua opinião, a variolização só podia ser realizada com certa experiência. Havia um período propício para a extração do pus, cuja técnica incisiva impedia a saída de grande quantidade de sangue; caso contrário, o material seria contaminado e, consequentemente, descartado (Cabussú, 16 abr. 1863).

Muitos médicos brasileiros da segunda metade do século XIX alegavam que os problemas técnicos relativos à vacinação eram decorrentes da ausência de formação médica dos vacinadores municipais. A falta de conhecimento poderia levar os vacinadores a confundir as pústulas falsas, surgidas em indivíduos já imunizados, com as verdadeiras. O material retirado dessas pústulas falsas seria ineficaz em futuras inoculações. Para tentar se esquivar da acusação de ineficácia da vacina, os médicos responsáveis pelos serviços de vacinação alegavam que surtos de varíola eram confundidos com outras doenças como varioloide ou catapora, conhecidas como “bexigas doudas”. Argumentavam ainda que a variolização, por ser praticada no Brasil simultaneamente com a vacina, confundia a população leiga, considerada incapaz pela comunidade médica de distinguir os dois métodos (Chalhoub, 1996).

Conforme vimos, Cabussú enfatizou a importância da separação dos doentes e afirmou que os espaços de cura no Paraná não tinham uma distribuição espacial mais rígida e racional que impedisse a disseminação de surtos epidêmicos. Esse esquadrinhamento dos indivíduos enfermos e sadios é uma técnica disciplinar aplicada com o objetivo de garantir uma determinada segurança no âmbito dos hospitais (Foucault, 2008). Cabussú ainda criticou a livre circulação da tripulação do iate pelo interior da urbe de Paranaguá. Aqui podemos observar outro aspecto de tipo disciplinar relativo ao regulamento da circulação de indivíduos que nos remete a uma prática bastante controversa durante o século XIX, isto é, as quarentenas. Também é possível identificar os elos que unem as questões relacionadas à circulação com os problemas urbanos, mais especificamente, com a cidade. Assim, ela é pensada como uma forma de organizar e gerir um acúmulo ou um grupo de indivíduos. Doenças epidêmicas, crises de abastecimento, violência generalizada comprometeriam algumas das principais funções de uma urbe, tais como o ordenamento da circulação e a coexistência pacífica entre seus habitantes.

A respeito do surto de varíola ocorrido em Paranaguá em 1863, Pedro de Araújo Lima, o marquês de Olinda, então ministro e secretário de Estado dos Negócios do Império, enfatizou em seu relatório a diferença entre a varíola espontânea ou natural e a vacina humanizada oriunda do *cowpox*. Conforme assinala Araújo Lima, na varíola natural havia apenas uma única erupção, precedida por febre fugaz. A varíola inoculada (vacina) seria caracterizada por duas erupções distintas: uma primitiva e local, cuja manifestação ocorria geralmente ao fim do terceiro dia de moléstia, e outra, mais geral e tardia, anunciando-se por uma rápida febre. As cicatrizes também forneciam indícios importantes para o diagnóstico, segundo o marquês. A supuração das pústulas de varíola espontânea causava estrias profundas e indeléveis. A inoculada produzia cicatrizes superficiais, pois as pústulas não chegavam a verter pus. Elas seriam profundas apenas nos locais da incisão, onde se desenvolvia leve supuração (Lima, 12 jun. 1863).

Araújo Lima mostrou-se preocupado diante dos relatórios de Bousquet e Cabussú. Considerou-os confusos, desordenados e excessivamente concisos, impossibilitando a extração de conclusões científicas cabais. Havia muitas omissões, o que poderia revelar, segundo o marquês, negligência nos exames; ocultação da verdade ou espírito pouco habilitado em coligir dados indispensáveis para a história de uma epidemia. Em seu relatório, o ministro do Império acusa Bousquet de não detalhar em suas exposições as diferenças entre os sintomas da variolização e da vacina. A mudança de diagnóstico proferida pelo médico de Paranaguá, mencionada anteriormente, reforça a hipótese do ministro da falta de expertise, muito embora o processo imunitário e o agente viral da varíola fossem desconhecidos àquela época.

O vacinador de Paranaguá inoculou duas turmas, uma no dia 8 e outra no dia 15 de abril de 1863. Porém, nem Bousquet nem Cabussú souberam especificar quantas crianças de cada grupo foram inoculadas. Bousquet, que chegou à cidade no dia 16 do respectivo mês, não informou à qual grupo pertenciam os enfermos por ele observados. Para Araújo Lima, se esses enfermos foram inoculados no dia 15, os sintomas não poderiam ser constatados no dia seguinte, sendo, portanto, fácil deduzir que já possuíam o germe incubado. Nesse caso, o diagnóstico de febre eruptiva variólica, ou bexigas naturais, faria sentido.

Araújo Lima considerava plausível a hipótese de que a varíola chegara a Paranaguá a bordo do iate e fora espalhada pelo interior da cidade mediante os cinco trabalhadores responsáveis pela descarga da embarcação. Ele discorda de Cabussú ao não ver como causa do surto a negligência do médico encarregado pelas visitas no porto ou a inoculação realizada pelo vacinador municipal. Ao concluir seu relatório, o ministro ressalta a importância de ouvir a versão desses dois profissionais a respeito dos fatos ocorridos (Lima, 12 jun. 1863). Em nota manuscrita à margem esquerda do documento, lê-se que a discussão técnica não interessava ao governo. O problema consistia em saber se o surto fora causado pelo descaso do inspetor, acusado de não ter feito inspeção rigorosa no iate. É muito provável que as autoridades estivessem relutantes em assumir como uma das principais causas do flagelo a variolização praticada pelo vacinador.

Vejamos a seguir as características do primeiro regulamento para vacinação da província do Paraná criado pelo médico José Cândido da Silva Murici. Murici estabeleceu critérios para a formação de mapas estatísticos, buscou compreender as razões pelas quais a aceitação da vacina era limitada e realizou as estatísticas dos vacinados entre 1856 e 1863.

## Regulamento de vacinação e mapas estatísticos

O primeiro regulamento para a vacinação na província do Paraná, com seis artigos, foi elaborado pelo comissário vacinador provincial José Cândido da Silva Murici e publicado em 1863. De acordo com o primeiro artigo, todos eram obrigados a se vacinar, independentemente de idade, sexo, estado ou condição. Aqueles que se recusassem deveriam ser punidos conforme as posturas municipais. A responsabilidade pelos escravos e menores era dos respectivos senhores, pais ou tutores. Após oito dias, os vacinados deveriam retornar aos locais de vacinação para a extração do fluido vacínico (visto que o Brasil não produzia a *cowpox*), que seria armazenado em lâminas e tubos de vidro para futuras tentativas de imunização. Pessoas que não apresentassem qualquer reação à vacina deveriam retornar após três meses para uma nova tentativa de imunização. Se, mesmo assim, a vacina ainda não surtisse efeito, deveriam retornar três anos depois, salvo se nesse intervalo ocorresse alguma epidemia (Paraná, 1863).

O artigo dois determinava que os vacinadores possuíssem cadernos regularmente escriturados para anotar nome, filiação, idade, estado e condição do vacinado. Deveriam ainda assinalar aqueles em que a vacina não teve efeito, que não retornaram para a extração do fluido ou para nova tentativa de vacinação. Os vacinadores também tinham que registrar os indivíduos que se recusaram a receber a vacina, informar seus nomes às Câmaras Municipais e às autoridades policiais, e detalhar as medidas adotadas para a aplicação das penas e a data em que essas medidas foram executadas (Paraná, 1863).

O quarto parágrafo do artigo dois e o artigo cinco determinavam, respectivamente, a obrigação dos vacinadores de elaborar mapas estatísticos dos vacinados a cada três meses e manter uma relação nominal das pessoas residentes nas circunscrições territoriais sujeitas à sua ação administrativa. Para isso, deveriam solicitar dados estatísticos sobre a população aos párocos e autoridades policiais (Paraná, 1863).

O regulamento ainda estabelecia aos vacinadores a mais escrupulosa vigilância em relação a tudo que pudesse interessar ao serviço de vacinação e contribuir para generalizar a vacina e ampliar a sua eficácia. Isso incluía, conforme os parágrafos cinco, seis e sete do artigo dois, o fornecimento de certificados aos vacinados, o máximo de cuidado possível com a conservação dos fluidos vacínicos e a notificação imediata de casos de varíola (Paraná, 1863).

De acordo com José Cândido da Silva Murici, o regulamento melhorou significativamente os serviços de vacinação, ampliando a difusão da vacina nas localidades mais afastadas e diminuindo a oposição a ela. Essas afirmações podem ser comparadas com as estatísticas computadas pelo próprio Murici, que revelam um aumento de 511% no número de lancetados entre o período de 1856 e 1863 na província do Paraná (Rohan, 1856; Carvalhaes, 1858; Nogueira, 1863; Silva, 1864). Mas é preciso cautela ao olhar para esses dados. O aumento ocorreu de forma exponencial em 1862 e 1863, justamente durante o surto que grassou em Paranaguá, quando 234 pessoas foram vacinadas na cidade. Em 1864, esse número caiu para 157 (Fleury, 1865). Isso era uma característica observável em outras províncias, isto é, a procura pelo imunizante aumentava somente em épocas de epidemia. De toda forma, parte da população paranaense buscou os recursos da medicina acadêmica na esperança de evitar o contágio, ensejando a hipótese segundo a qual a resistência à vacina não configurava um fenômeno generalizado e sedimentado.

O que significava a realização de um levantamento estatístico sobre um determinado fenômeno mórbido no Brasil da segunda metade do século XIX? Na França, a institucionalização das técnicas da estatística na medicina ocorreu durante a década de 1830 mediante ação de alguns higienistas. O objetivo deles era estabelecer uma carta nosográfica do globo terrestre. O método estatístico já vinha sendo utilizado por Philippe Pinel (1745-1826) na primeira década do século XIX, mas a generalização na prática médica só ocorreria de fato entre as décadas de 1820 e 1830 (Edler, 2011).

Informações como o número de infectados, faixa etária, etnia, gênero, condição social, região, auxiliam, entre outros, na compreensão e elaboração de estratégias de controle das doenças que afetam não apenas indivíduos, mas o equilíbrio e o funcionamento de sociedades. Esses dados possibilitam, ainda, a formação de um saber relativo a uma das riquezas mais importantes do Estado, isto é, a população, sobretudo quando ela é considerada um fator de produção.

Sem uma população saudável e segura, não há circulação de pessoas e, por conseguinte, desenvolvimento econômico e recursos para a manutenção de um aparato estatal. Certamente, as taxas de morbidade por patologias, assim como as taxas de criminalidade e carestia, por exemplo, dificilmente atingem níveis próximos de zero em alguma sociedade. É possível conjecturar que isso não seja desejável do ponto de vista de algumas teorias econômicas. Há um limite tolerável para essas taxas, e as estatísticas seriam o meio mais adequado de mensurar esse limite, possibilitando aos agentes estatais intervir com mais precisão no corpo social (Foucault, 2008).

No que diz respeito às estatísticas computadas por Murici, além desses aspectos, elas representaram um esforço no sentido de constituir um conhecimento relativo tanto à varíola como à população paranaense. Esse esforço se somava aos de outros médicos em outras províncias do Império. Sem essas informações, não seria possível modernizar o aparato administrativo das instituições do Estado brasileiro e formular políticas de saúde pública coerentes e profícuas, sobretudo em uma conjuntura histórica onde as pressões internas e externas exigiam a integração do país aos circuitos mundiais da economia capitalista.

Nomeado Comissário Vacinador Provincial em 1854, José Cândido da Silva Murici buscou compreender a limitada aceitação da vacina. Entre os principais motivos apontados por ele estavam: a inexistência de um regulamento capaz de instruir os vacinadores e esclarecer a população; a descrença dos habitantes no preservativo vacínico; a extraordinária extensão territorial do país e, em menor medida, da província paranaense, somadas à deficitária estrutura viária nacional; a falta de fiscalização dos preceitos legislativos determinados pelas posturas municipais; a falta de remuneração aos comissários vacinadores; e, por último, mas não menos importante, os inúmeros problemas técnicos e operacionais relativos à importação do pus vacínico e às dificuldades de solicitação do fluido junto ao Instituto Vacínico da Corte (Silva, 1864). Todas essas questões podiam ser observadas em outras províncias do Império. Em essência, é possível afirmar que esses fenômenos atingiam o país como um todo, conferindo uma característica singular aos serviços de vacinação antivariólica no Brasil imperial.

## Considerações finais

O conjunto de documentos consultado para a elaboração deste artigo revelou os esforços e as dificuldades de disseminação da vacina antivariólica na província do Paraná ao longo da segunda metade do século XIX. As fontes evidenciam a existência de um consenso praticamente unânime entre a comunidade médica e as autoridades públicas daquela época, segundo o qual o fracasso da generalização da vacina era devido à ignorância popular. A despeito disso, essa mesma documentação indicou a atuação de populares que pressionaram os médicos de Paranaguá pela aplicação do imunizante, relativizando a tese da resistência popular. Havia resistência, mas ela não era generalizada. Mesmo assim, as autoridades públicas argumentavam sobre a necessidade de medidas disciplinares mais severas com o intuito de fazer cumprir rigorosamente as medidas estabelecidas pelas posturas municipais a respeito da vacinação. Ministros e presidentes de província propugnavam a ampliação dos poderes estatais no âmbito da saúde pública para melhorar os índices relativos à vacinação. Essa discussão insere o debate historiográfico sobre as medidas sanitárias adotadas por Oswaldo Cruz e a revolta da vacina no Rio de Janeiro, em 1904, em uma perspectiva histórica mais ampla e de longa duração.

A documentação também apontou a existência de outras questões igualmente complexas relacionadas à difusão da vacina no Brasil oitocentista. Essas questões diziam respeito aos obstáculos para a produção e distribuição do imunizante em um país com extraordinária extensão geográfica e uma rede precária de ferrovias e rodovias. Mesmo com um dispositivo disciplinar mais coercitivo, a infraestrutura e os recursos disponíveis na época não permitiriam uma distribuição rápida e segura do imunizante para regiões distantes dos principais centros urbanos do país. A infraestrutura deficitária e a limitação de recursos, que caracterizavam de forma singular os processos de circulação no Brasil imperial, impunham desafios contundentes às autoridades sanitárias desses espaços remotos. Existiam desequilíbrios de poder que distinguiam esses locais tidos como “periféricos”, mas que, na verdade, integravam uma rede mais complexa, não estática, com suas singularidades, dinâmicas próprias e múltiplas conexões.

Uma dessas conexões, considerada um caso de circulação de curta distância e que revela as interações em níveis regionais, foi a atracação de um iate oriundo de Santa Catarina no porto de Paranaguá com tripulantes acometidos pela varíola. O episódio foi considerado pelos atores envolvidos no “teatro de acontecimentos” como a origem do surto de varíola que atingiu a cidade em 1863. A despeito das acerbas controvérsias científicas a respeito da propagação inicial do surto, se a partir do iate ou da prática equivocada da variolização, esse acontecimento trouxe à tona a especificidade dos serviços de vacinação no Império e os embaraços relativos à importação e à distribuição da vacina. Esses entraves demonstram como os processos de circulação no Brasil imperial não eram suaves, naturais, padronizados e homogêneos. Em outros termos, recursos técnicos, científicos, financeiros e humanos circulavam de maneira distinta entre as diferentes províncias. No Paraná, assim como em outras regiões do país, faltava vacina e vacinadores em função das baixas gratificações pagas. Não havia dinheiro ou vontade política para o aumento dessas gratificações, para o treinamento desses vacinadores e para ampliação da estrutura viária. Essas dificuldades não foram consideradas aqui simplesmente como problemáticas, mas como constituintes do próprio processo de circulação de uma prática: a vacinação.

Por fim, vimos como José Cândido da Silva Murici utilizou as estatísticas médicas para compilar dados referentes à vacinação antivariólica no Paraná. Foram examinadas as relações existentes entre essas estatísticas e a formação de um conjunto de informações referentes a uma determinada população a ser governada. Em uma conjuntura marcada pelo avanço do capitalismo industrial e a necessidade crescente de mão de obra adestrada e saudável, a população passou a ser cada vez mais esquadrinhada e, sobretudo, assistida. Evitar o aumento das taxas de criminalidade, carestia e mortalidade, bem como a propagação de doenças epidêmicas, e assegurar os processos de circulação cruciais para a manutenção das populações tornaram-se imperativos para que os países da América Latina pudessem modernizar suas estruturas estatais e, assim, integrarem-se aos circuitos da produção mundial capitalista e ao rol de nações consideradas civilizadas.

## NOTAS

1 Diante da impossibilidade de arrolar neste espaço toda a bibliografia produzida no Brasil e no exterior a respeito da história da varíola, optamos por selecionar alguns dos trabalhos cujas contribuições consideramos fundamentais para o debate historiográfico. Em relação às reflexões que abordam a resistência popular à vacina sob os aspectos culturais, sociais, políticos e econômicos, destacamos: Costa (1986); Carvalho (1987); Sevcenko (1993); Chalhoub (1996). Para uma abordagem da doença no período colonial brasileiro, consultar: Campos (1956); Alden, Miller (1987); Chambouleyron et al. (2011). No que concerne a análises circunscritas ao século XIX e à primeira metade do século XX, sugerimos: Fernandes (1999); Cueto (2004); Silveira, Marques (2011); Soumonni (2012); Silveira (2013); Almeida (2014); Agostini (2015). Sobre a erradicação da varíola, indicamos: Hochman (2009); Hochman, Palmer (2010). Para estudos que examinam controvérsias científicas relacionadas à varíola, ver: Teixeira (2000); Caponi (2002); Fernandes (2003). Sobre a análise iconográfica das campanhas de imunização, consultar: Porto, Ponte (2003).

2 Em 1854, a Província do Paraná contava com uma população de aproximadamente 62 mil pessoas (Vasconcelos, 1854).

3 Na segunda metade do século XIX, a medicina acadêmica não exercia um monopólio do mercado terapêutico no Brasil, pois, além da desconfiança popular em relação aos alopatas, ela não apresentava resultados promissores no que diz respeito à cura de enfermidades. Isso levava muitas pessoas pertencentes aos estratos mais baixos da sociedade a recorrer a métodos heterodoxos de cura. Sobre a rivalidade entre distintas racionalidades terapêuticas no Brasil, consultar Pereira Neto (2001).

4 Sobre as artes de curar e a atuação dos curandeiros no Brasil, consultar Pimenta (1998, 2004); Witter (2015).

5 Sobre as origens da varíola e seus sintomas, assim como as diferenças entre variolização, vacina jenneriana e vacina animal e a introdução dessas práticas ou técnicas no Brasil, consultar: Rosen (1994); Chalhoub (1996); Fernandes (1999, 2003); Gurgel (2010); Hochman, Souza (2022). Controvérsias em torno do diagnóstico da varíola ainda podiam ser observadas no Brasil na década de 1930. Sobre essa questão, ver Teixeira (2000).

6 Sobre a construção das primeiras ferrovias no Brasil, consultar Graham (1973), principalmente o capítulo 2.
